# Australian Alpaca Demographics and Management: A National Survey

**DOI:** 10.3390/ani14192861

**Published:** 2024-10-04

**Authors:** Imogen Boughey, Evelyn Hall, Russell Bush

**Affiliations:** Sydney School of Veterinary Science, The University of Sydney, 425 Werombi Road, Camden, NSW 2567, Australiarussell.bush@sydney.edu.au (R.B.)

**Keywords:** alpaca, demographics, survey, farm production, animal management

## Abstract

**Simple Summary:**

The Australian alpaca industry is known for fibre production. Despite the growth of alpaca numbers since their introduction in the 1980s, little is known about their distribution or on-farm management practices in Australia. This study used an online survey to gain insight into the current demographics, animal management practices and key knowledge gaps. Of the 88 respondents, the majority were located in the high-rainfall areas of the east coast of Australia, which could be due to consistent year-round pasture availability and market access opportunities. Of the two alpaca breeds, the Huacaya accounted for 93% of the animal numbers reported in this survey. Twelve key pasture species were identified, with Kikuyu the most common, followed by Subterranean Clover and Phalaris, likely due to the high concentration of respondents in suitable high-rainfall environments. Pasture species were not identified by 25% of respondents. The off-label use of veterinary chemicals for disease and parasite control resulted in a variation in dosage rates and administration frequency, raising concerns for effective management. These results highlight important knowledge gaps in nutritional and health management practices that require further research and practical industry recommendations to improve alpaca health and productivity.

**Abstract:**

The Australian alpaca industry has continued to grow since the introduction of alpacas in the 1980s. Little is known about the geographical distribution of alpacas or on-farm management practices. This study aimed to address this and identify key producer knowledge through an online survey. The survey consisted of 25 questions grouped into 3 areas: demographics, farm production and alpaca nutrition. The highest concentration of alpaca producers was along the east coast of Australia, primarily in high-rainfall zones, which could be attributed to more consistent year-round pasture availability and market access opportunities. The Huacaya breed accounted for 93% of the animal numbers reported in this survey. Respondents identified 12 key pasture species being grazed, with Kikuyu being the most common, followed by Subterranean Clover and Phalaris, likely due to the majority of respondents being located in suitable high-rainfall environments. Pasture species were not identified by 25% of respondents. There are no registered anthelmintics or vaccinations for alpacas, resulting in a variation in dosage rates and administration frequency, raising concerns for effective disease and parasite management. This survey has identified key knowledge gaps in alpaca management practices in Australia that will be further investigated to provide industry recommendations to improve alpaca production.

## 1. Introduction

The Australian alpaca industry began with importations from South America in the 1980s to 1990s to develop an alternative animal fibre industry [1,2]. The Australian alpaca industry has continued to grow in Australia with an approximate current population of 350,000 animals spread across the temperate regions of Australia [3]. Previous survey studies into the Australian alpaca industry have focused on worm control [4], the management of other common diseases such as vitamin D deficiency and staggers [5,6,7,8], fibre and meat production [2,9,10,11,12,13] and an overview of smallholders and animal health management across various livestock industries [14]). There has been no previously published general demographic or farm management survey data for the alpaca industry in Australia. Improving livestock productivity can lead to increased farm income and business profitability [15,16]. However, as nutrition and herd management are key factors impacting livestock productivity, limited information for Australian-raised alpacas creates a barrier for future improvement [15]. Improving the productivity and sustainability of the Australian alpaca industry requires identification of current practices and issues. Alpaca management practices in Australia are often derived or extrapolated from recommendations for sheep, including worm control [4,17], vaccination [7] and nutrition and feeding guidelines [18,19]. Prior to 2019, there was no demographic information on the Australian alpaca industry. Rashid et al. [4] included demographic questions in an industry survey in 2019 to determine internal parasite control practices in Australian alpacas. An average herd size of 57 alpacas was determined, with 67% of producers keeping the Huacaya breed [4]. It is important to establish updated data since 2019 to establish the industry’s growth and identify issues that have arisen to provide informed research and resources to improve industry productivity. This study aimed to provide current industry demographics on the Australian alpaca industry and identify common management practices and issues. This advanced understanding of current management practices will enable alpaca-specific guidelines that improve productivity to be developed.

## 2. Materials and Methods

### 2.1. Survey Participants

The survey was open to any individual or entity owning alpacas in Australia with participation optional. The survey was distributed through industry channels, where members agreed to receive correspondence, as well as social media. The survey was also promoted at industry events, including Agricultural Shows, where the survey electronic link was provided to interested producers. All data collected from submitted responses were non-identifiable, with participants submitting the postcode where their alpacas reside to determine geographical spread. Human ethics approval was granted by The University of Sydney Human Ethics Committee (project number 2022/720).

### 2.2. Survey

The survey was conducted using the Microsoft Forms online platform. The survey comprised 35 questions grouped into 3 key areas: alpaca nutrition, farm production and farm demographics. Of the questions,10 were close-ended with several options, 7 were semi-open-ended (e.g., with an ‘other’ option), and 15 were open-ended where providing options was not suitable (e.g., a numerical answer was required). The alpaca nutrition section contained questions on animal health and management to identify common procedures, nutrition, and key animal-related issues. In this section, respondents were asked to select their ideal body condition score (BCS) for different production classifications. BCS is ranked between 1 and 5, with 1 being severely underweight and 5 obese, as BCS is a reflection of nutritional status [20]. Vaccination frequency was divided into 4 categories for statistical analysis; 4 times a year, every 6 months, yearly and never. The frequency of veterinary visits was split into less than once per month, 1 to 5 visits per month, 1 to 5 visits per year. The farm production section contained questions regarding land management, including stocking rate and key pasture species. Stocking rate was measured in number of alpacas per hectare. The farm demographic component focused on the location of properties and herd qualities including breed, sex and animal number. Respondents were asked to identify their production conditions based on annual rainfall amounts. High-rainfall zone areas receive >600 mm annual rainfall, sheep/wheat zone receives 300–600 mm rainfall and pastoral zone <300 mm rainfall.

### 2.3. Statistical Analysis

Analyses were performed using R Version 4.4.1 [21] and Microsoft Excel 2018 [22]. Survey data was downloaded from Microsoft Forms as an Excel (xlsx) file before being formatted and saved as a comma-delimited (CSV) file to be imported into R (R Core Team, Vienna, Austria, 2022). Farms were categorised in herd size based on the animal numbers provided. Small scale included farms with up to 49 alpacas, medium-scale farms with 50 to 249, and large-scale farms with more than 250 alpacas. These brackets were chosen based on industry structure and similarities within the husbandry and management practices of farms within those brackets. For each variable, descriptive statistics were carried out using Excel [22]. Linear models were used to determine the effect of the production zone on the stocking rate. Generalised linear regression (GLR) models were conducted to assess the impact of production zone and herd size on vaccination frequency, seasonal ill thrift ranking (season in which highest rate of ill thrift occurs), stocking rate, pasture species, supplement types, and frequency of veterinary visits. A *p*-value of <0.05 was considered significant.

## 3. Results

The database contained 90 responses; 2 responses did not agree to participate and were excluded from the survey, leaving 88 responses.

### 3.1. Demographic Information

**Figure 1:**
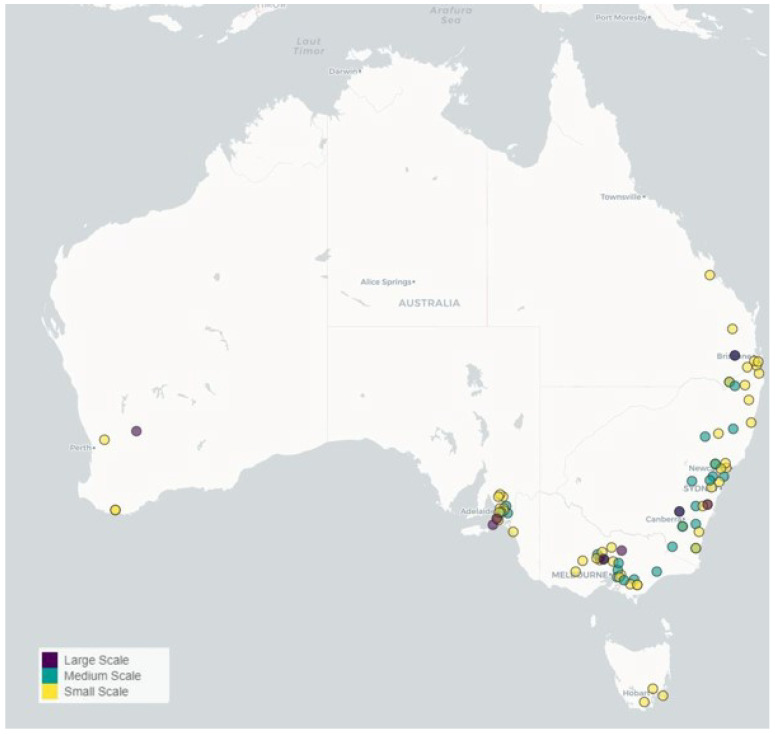
The location of respondents based on postcode. Colours depict farm scale. Small scale ≤49 alpacas, medium scale 50–249 alpacas, large scale ≥250 alpacas.

New South Wales (NSW) recorded the highest number of responses, with 35.2% of respondents, followed by Victoria (VIC) with 27.3%, South Australia (SA) 17.0%, Queensland (QLD) 12.5%, Western Australia (WA) 4.5% and Tasmania (TAS) 3.4% (Table 1). There were no respondents from the Northern Territory or the Australian Capital Territory The majority of respondents were located along the east coast of Australia (Figure 1). As the farm scale increases, the properties are located further inland, with the exception of South Australia (Figure 1). The high-rainfall zone (>600 mm) was selected by 52.3% of respondents, followed by 37.5% in the sheep/wheat zone (300–600 mm) and 10.2% of respondents located in the pastoral zone (<300 mm). NSW has the highest number of small- and medium-scalefarms followed by VIC. However, VIC reported three large-scale farms compared to two in NSW.

The primary reason for farming alpacas in Australia is for small-scale production or as a smallholder (alpacas are not the primary income source) (69%). Commercial production for fibre was selected by 12% of small-scale producers and 77% of large-scale producers. Stud/Animal Sales were the primary focus for 10% of respondents, with 55% classified as medium scale. The two responses, in other, did not provide enough information to be included in a category. Reasons for primary production are presented in Table 2. Additionally, only 10% of respondents supplied animals for meat.

Huacayas accounted for 93% and Suri 7% of the animal numbers reported in this survey. Of the respondents, 92% own Huacayas and 48% own Suri. Furthermore, 60.3% of respondents owned only 1 breed, 52.3% only Huacays and 8% only Suri.

### 3.2. Farm Management

**Figure 2:**
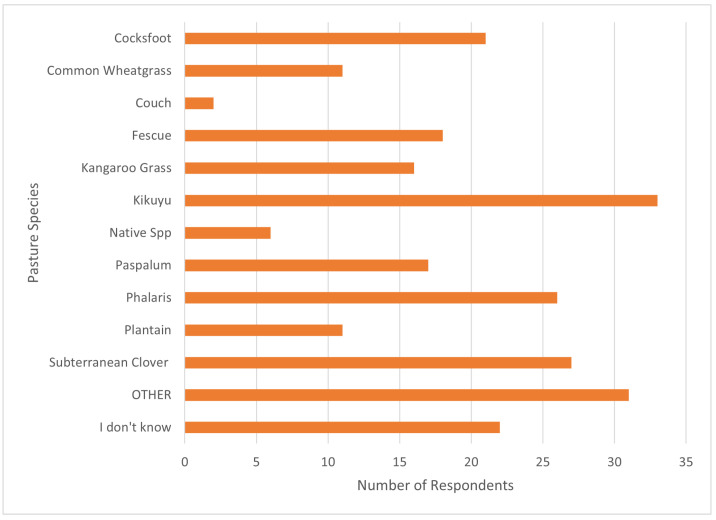
Identified pasture species grazed by alpaca producers throughout the year.

**Figure 3:**
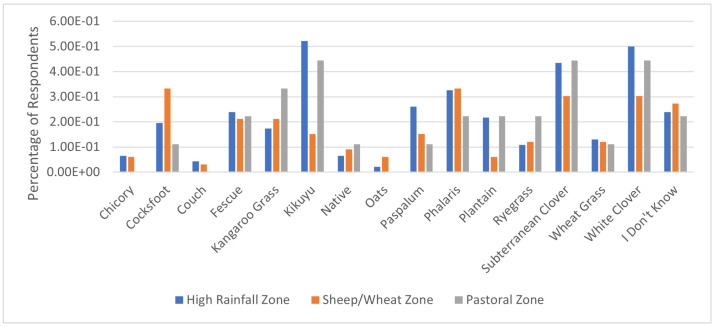
Predicted pasture species occurrence by production zone.

Respondents identified 12 key pastures used in their grazing systems. Kikuyu was the most common pasture being grazed (37.5%), followed by subterranean clover (30.7%) and Phalaris (29.5%) (Figure 2). Pasture species could not be identified by 25% of respondents. Production zone impacted the occurrence of Kikuyu (*p* < 0.05) with 52.1% (±7%) of farms in high-rainfall zones growing Kikuyu compared to 15.2% (±6%) of sheep/wheat zone farms (Figure 3). White clover was more likely to be grown in high-rainfall zones compared to both the sheep/wheat and pastoral zones (Figure 3).

The stocking rate was recorded as the number of alpacas per hectare. Stocking rate in the high-rainfall zone was 5.5 ± 0.53, the pastoral zone was 7.21 ± 1.29 and the sheep/wheat zone was 3.77 ± 0.63. Stocking rate was significantly affected by production zone (*p* = 0.04). There was no significant difference between the high-rainfall zones and pastoral (*p* = 0.31) or sheep/wheat zone (*p* = 0.22). There was a significant difference in stocking rate between the pastoral and sheep wheat zones (*p* < 0.05), with an estimated difference of 3.44 ± 1.44 (SE) alpacas per hectare. There was no significant difference between the seasonal occurrence of ill thrift and production zone for all seasons (summer [*p* = 0.29], autumn [*p* = 0.36], winter [*p* = 0.67], spring [*p* = 0.29]). Winter was most commonly selected as the season when ill thrift and weight loss was most likely (45.45%), and spring least likely (7.95%).

### 3.3. Animal Management

**Figure 4:**
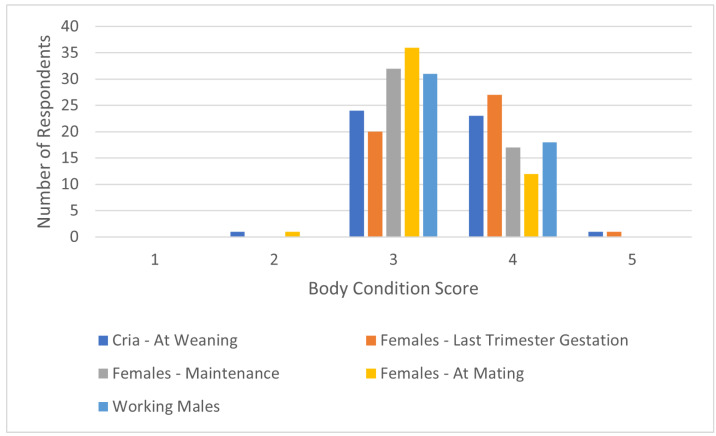
Ideal alpaca body condition score (BCS).

Forty-nine respondents completed the BCS answers for the categories ‘Cria—At Weaning’, ‘Females—Maintenance‘, ‘Females—At Mating’, and ‘Working Males’. There were 47 responses for ‘Females—Last Trimester Gestation‘. The majority of respondents selected three or four as the ideal BCS for most categories (Figure 4).

**Figure 5:**
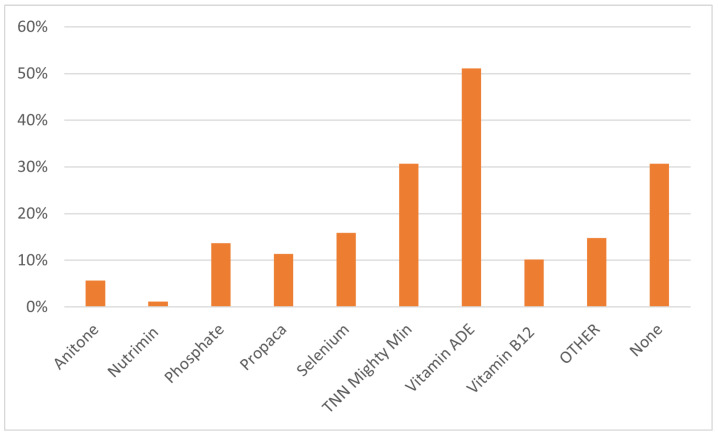
Percentage of respondents providing regular additional supplements for alpaca management.

Fortified pellets were fed by 48.2% of respondents. Alpaca pellets were fed by 88.6% of respondents who fed any pellets, horse pellets by 9.1% and cattle pellets by 2.3%. Neither herd size (*p* = 0.63) nor production zone (*p* = 0.10) significantly impacted whether respondents fed pellets. Respondents were asked if they regularly supplemented with the following commonly used supplements; comprehensive liquid supplement (containing vitamins, minerals and amino acids), vitamins A, D and E, selenium, phosphate, basic liquid supplement, energised nutrition supplement formulated for Australian camelids, vitamin B12 or no additional supplements. Production zone had no significant effect on administering supplements (all *p* > 0.05; Table 3). Herd size significantly impacted supplementing with selenium (*p* = 0.0238), but had no effect on any other supplements included in the survey (Table 3). Vitamin A, D and E were the most commonly administered supplements used by 51.1% of respondents, followed by basic liquid supplements and no supplement provided (30.7% of respondents) (Figure 5).

**Figure 6:**
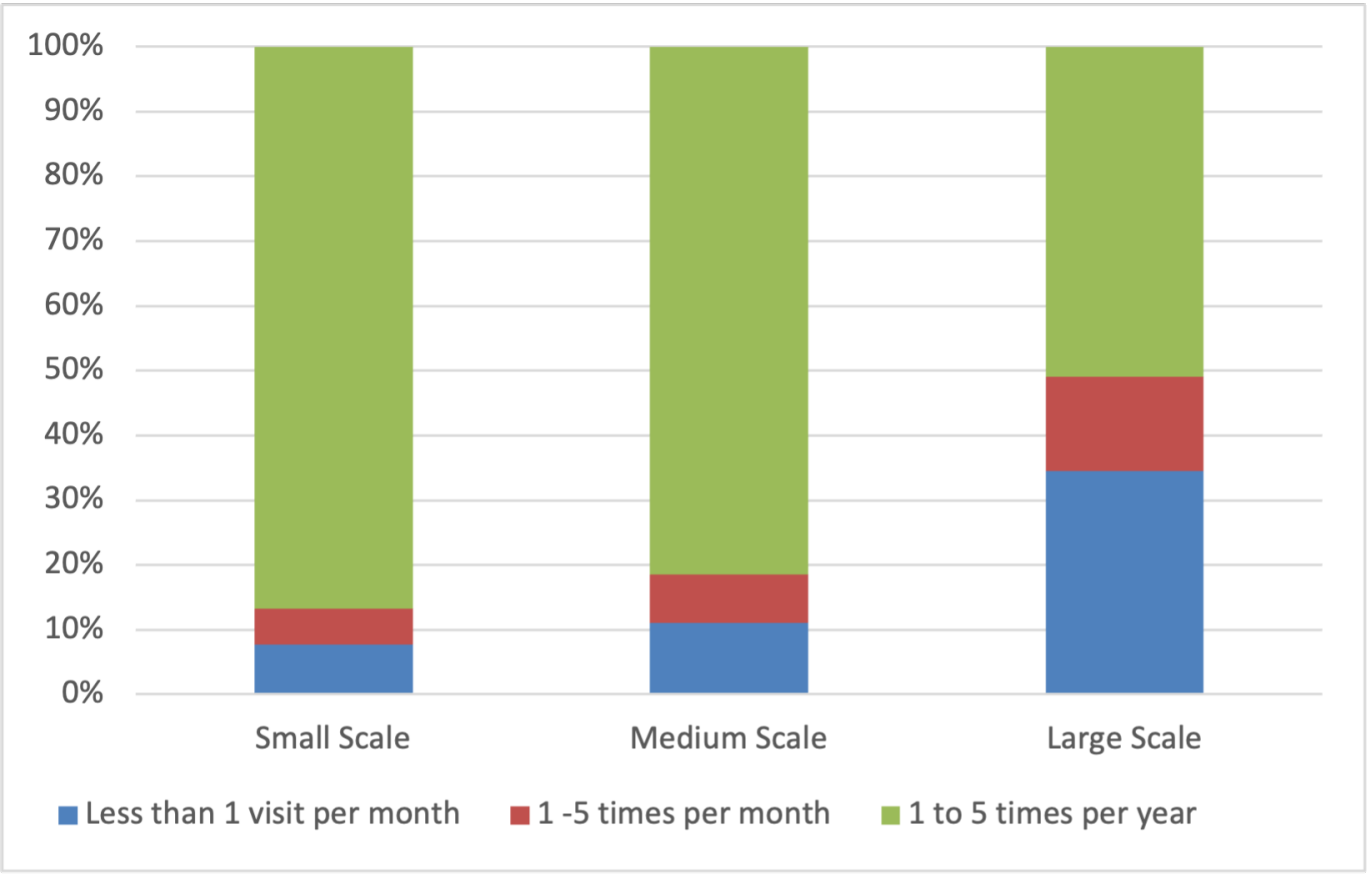
Percentage of respondents providing regular additional supplements for alpaca management.

The frequency of veterinary visits was not significantly impacted by herd size (*p* = 0.13). However, there was an increased frequency of visits as herd size increased (Figure 6). Birthing complications was the highest-ranked reason for veterinary visits for 43.18% of respondents, followed by external injury for 22.73% (Table 4).

**Figure 7:**
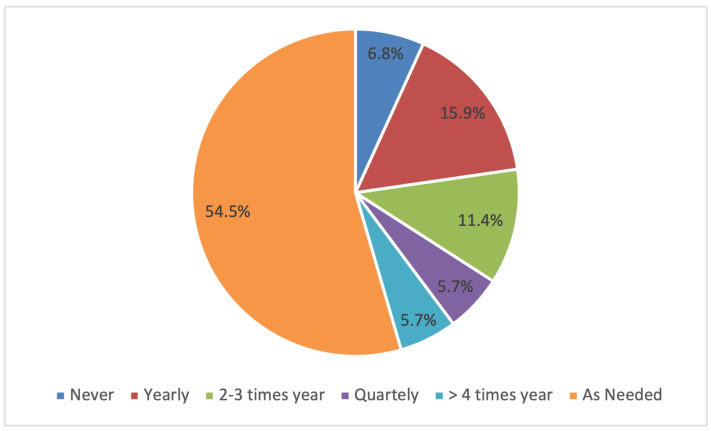
Frequency of drenching alpacas for worm treatment and prevention.

The frequency of drenching for worm prevention or treatment ranged from never to over four times per year, with 55% of respondents identifying that they drench as needed (Figure 7). Of the 48 respondents that drenched as required, 52% noted that they drenched based on a faecal egg count assessment. Respondents that drench as needed also included weather conditions as a key deciding factor, with a trend to increase drenching frequency during increased rainfall. There was 7% of respondents who identified that they never drench the alpacas on their property (Figure 7). Overall, 45 out of 88 respondents identified that they conduct faecal egg counts (FEC) prior to drenching, 12 respondents sometimes conduct FECs and 32 respondents do not conduct FEC. There were no significant effects of the production zone on drenching frequency. Respondents were asked to identify what signs and symptoms they look for before drenching a sick animal: 55% of respondents included descriptions of pale mucous membrane and anaemia symptoms, 50% of respondents considered weight loss or reduced body condition, 30% looked at changes in faecal consistency such as scouring and 40% take into account changes in behaviour, movement and energy levels prior to drenching. Of the respondents that drench their animals, 22% only identified one factor that they looked at prior to drenching; 12% conducted FEC, 5% assessed gum colour, 2% assessed weight or body condition loss, 1% considered changes in animal movement and 1% assessed fecal consistency.

**Figure 8:**
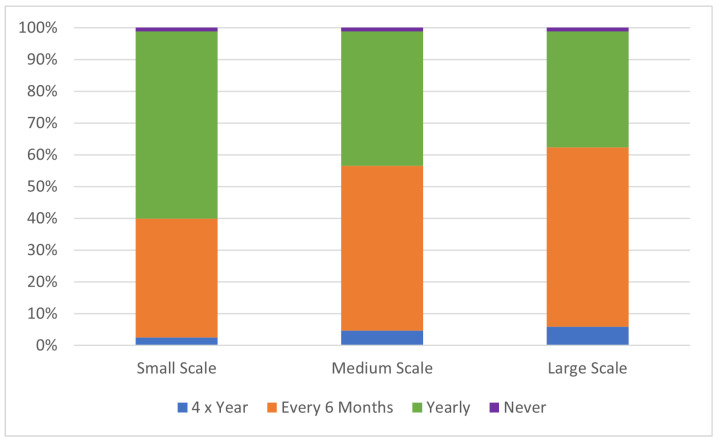
Frequency of vaccination of alpacas based on herd scale.

Herd size did not significantly impact vaccination frequency (*p* = 0.23). There is a trend towards increased vaccination frequency where herd size increases (Figure 8). Large-scale farms are approximately 2.65 times (95% CI, 0.66 to 11) more likely to have a higher rate of vaccination when compared to small-scale farms, while medium-scale farms are 2.1 times more likely Figure 8). The vaccinations identified by respondents were: 5in1 (clostridial diseases including enterotoxaemia, tetanus, black disease, malignant oedema and blackleg), 6in1 (clostridial diseases plus cheesy gland) and 7in1 (clostridial diseases plus leptospirosis). The 5in1 vaccination was the most selected (72.7% of respondents), followed by the 6in1 vaccination (22.7%). The 7in1 vaccination was chosen by 2.3%, and the 8in1 or no vaccination options were selected each by 1.1% of respondents. Yearly vaccination frequency was the most common, selected by 48.9% of respondents, followed by vaccination every 6 months (43.2%), 4 times per year (3.4%) and other routines including no vaccination or no regular use (4.6%).

## 4. Discussion

### 4.1. Demographics

The majority of the Australian alpaca survey respondents were located along the east coast of Australia, with smaller numbers in South Australia and Western Australia. This distribution was expected as Hernadez-Jover et al. [14] and Rashid and et al. [4] reported the distributions following a similar distribution. The higher concentration of alpaca producers on the East Coast could be attributed to more consistent year-round pasture availability as producers are in high-rainfall zones. It is also possible that alpaca producers have established enterprises on the east coast due to increased market accessibility, as most alpaca fibre-buying houses are located in NSW, VIC and QLD [23]. Huacayas continue to be the primary breed produced in Australia [4,24]. The popularity of Huacaya alpacas could be linked to the similarity in textile process and end uses as wool [25] and the large range of products available [26]. The number of producers owning both alpaca breeds has increased since 2019 [4], possibly due to increased interest in the Suri breed or a change in market opportunities for Suri fibre. In 2000, 80% of Australian alpaca herds contained less than 6 animals [27]; by 2019, 64% of herds contained less than 50 alpacas [4], and 60% in this study in 2023. In comparison, in a 2019 study by Hernandez-Jover and et al. [14] that also used the Australian Alpaca Association member’s database to distribute the survey, the proportion of smallholder farms (<50 animals) keeping alpacas as their primary income has increased from 1.7% to 12%. This change could be attributed to developments in international and domestic market opportunities, shifting the focus to businesses structured around commercial alpaca fibre production. Interestingly, this survey did not determine there was extensive use of alpacas as guard animals within sheep flocks, with only two respondents replying accordingly. This topic has been extensively covered in separate studies [28,29] and will not be addressed here. Currently, only 10% of respondents supply alpacas for meat production, which could be attributed to the few opportunities for animals to be commercially processed in Australia.

### 4.2. Animal Management

#### 4.2.1. Pasture Management

Twelve key pasture species were identified across Australia. Kikuyu was the most grazed pasture species in the survey. As Kikuyu is suited to the high-rainfall regions of eastern Australia, which have a higher proportion of alpaca producers, it is reasonable to conclude that climatic suitability contributes to the high use of this pasture [30,31,32]. Subterranean clover (subclover) is often included in a pasture mixture with Kikuyu to improve the nutritional quality of the pasture and provide feed outside of the Kikuyu or summer grass growing periods [32]. The number of respondents who could not identify what pastures they were grazing raises concerns for animal health and environmental management. Inadequate nutritional consumption can lead to a range of nutritional-based diseases, such as protein energy malnutrition (PEM) and mineral deficiency diseases [19,33]. In a pasture-based system, a knowledge gap in pasture identification can result in uninformed administration of supplements and treatment of nutritional deficiencies and diseases, as well as a reduction in feed utilisation impacting animal performance [19,33,34]. Grazing land management practices impact soil properties, including water infiltration, carbon sequestration and nitrogen use globally [35]. Lack of information can lead to inappropriate implementation of grazing practices, which negatively impacts soil health and animal performance, impacting overall farm productivity [34,35]

#### 4.2.2. Supplements

Although there is limited knowledge of alpaca nutrition and supplementation in Australia, it is well established that alpacas are at high risk of vitamin D deficiency when removed from their native high-altitude environment resulting in lameness, slow growth rate, stiff joints and inward-pointing knees [5,6,7,19,36,37]. This survey found that vitamin D was regularly supplemented via a combination vitamins A, D and E injection by 51.1% of respondents, which was higher than any other supplements identified in this survey. Vitamin D can also be administered through dietary supplements, which is an option that some producers may favour. There are Australian and international recommendations for vitamin D supplementations to prevent and treat vitamin D deficiency and associated disorders in alpacas and camelids [6,36,37]. This survey did not explore the reasons behind supplementation, and further research is required to confirm the reason for high levels of vitamin D supplementation by Australian alpaca owners. Some of the other supplements identified by this survey, including vitamin B12 and selenium, are administered based on a deficiency in the soil, pasture impacting dietary intake or presentation of symptoms, explaining the lower levels of regular supplementation [23,36]. Although the use of fortified pellets was common among survey respondents, there is minimal knowledge in the scientific and veterinary communities of the effectiveness and suitability of feeding pelleted feed to alpacas as a nutritional supplement [12,38]. Some respondents reported feeding horse pellets regularly. Although alpacas are not true ruminants, many of the nutritional recommendations and digestive processes are similar [18], which raises concerns about the suitability of feed formulated for horses being used in alpacas. Although horse feed contains key nutrition areas (protein and energy), ruminants and horses have different anatomical structures involved in consuming (dentition) and digesting (gastrointestinal tract) feed, resulting in different particle sizes and ingredients required for effective and safe absorption of nutrients [39]. The use of pelleted feed formulated for cattle also needs to be carefully managed, as feeding based on body weight may not account for metabolic differences between the species and result in nutritional imbalances [38]. Further research needs to be conducted to confirm the effectiveness of meeting nutritional requirements for body condition and fibre management through feeding commercial pelleted feeds and mixes formulated for alpacas as a supplement.

#### 4.2.3. Internal Parasite Control

Despite internal parasites, particularly gastrointestinal nematodes, causing significant health and production issues in alpacas in Australia and globally [40,41,42,43], there are no comprehensive guidelines for intestinal worm control or registered anthelmintic treatments [42]. Regardless of this, the majority of alpaca producers in this survey were practising intestinal parasite control. This survey found that 55% of respondents drenched without a regular schedule, with 52% basing their decisions on FECs. This amount has increased slightly from a 2019 survey, where 43% of respondents drenched as needed [42]. Comparatively, a 2021 survey of Australian sheep producers showed a different trend, with 74% following planned preventative treatments [44]. The frequency of drenching for producers following a schedule was varied, with more respondents drenching yearly (16%) and 2–3 times a year (11%) than quarterly (6%) or more (6%). In Australia, adult ewes, lambs and weaners, on average, were treated for worms 2.1 times/year [44]; goats were similar, being administered an anthelmintic on average 2.5 times/year [45], which is reflected in the trend seen in the alpaca industry. A common practice in sheep internal parasite control is spelling paddocks and moving stock to spelled or ‘cleaner’ pastures after drenching [44]. This practice was only performed by 23% of respondents in the study conducted by Rashid et al. [4], and additional methods of worm control were not looked at in this study. The difference in internal parasite management could be linked to the higher proportion of smallholder farms than commercial farms [14]. Internal parasite management guidelines and the registration of anthelmintic treatments for alpacas are important areas for future development to improve alpaca management and productivity.

#### 4.2.4. Vaccination

There are also no licenced vaccinations for use in alpacas. However, it is generally recommended by veterinarians and industry to use a 5in1 or 7in1 vaccination developed for sheep and cattle for the prevention of clostridial diseases and leptospirosis as these have been developed for similar livestock species (sheep and cattle) [46,47]. The “Code of Welfare for Alpacas and Llamas Australia, 2016” only contains a recommendation to vaccinate pregnant alpacas 4–6 weeks prior to unpacking or at 300 days of gestation with a 5in1 vaccination to support antibody levels in colostrum [46]. However, globally, recommendations are highly variable and often include initial vaccinations prior to/at weaning and annual boosters [33]. Across the world, the most common vaccination given to alpacas is to protect against Clostridial diseases [33,36,46,48,49]. The 5in1 vaccination was the most commonly used in this survey reflecting the key diseases identified by previous studies. The frequency of vaccination was significantly affected by herd size, with 1.1% of respondents indicating that they were not vaccinating compared to 6% in New Zealand in 2015 [49] and 12.2% of South American Camelid (SAC) owners in Germany in 2021 [36]. A recent UK survey of alpaca owners was similar and reported that 95.7% of respondents’ animals were vaccinated against clostridial diseases [50]. Overall, although there are minimal recommendations for alpaca-specific vaccination schedules, it is a common practice globally to maintain the health of alpaca herds. Future research in Australia is needed to confirm appropriate doses and vaccination schedules for alpacas to improve production, including reducing the need for excess veterinary chemicals where possible.

#### 4.2.5. Production Zone, Stocking Rate and Seasonal Variation

In this study, the production zone significantly influenced the stocking rate between the pastoral and sheep/wheat zones. This was expected due to general livestock management practices based on annual average rainfall and feed availability. In comparison, sheep are commonly grazed at a higher stocking rate (e.g., 14–24 DSE (dry sheep equivalent) in coastal regions with improved pasture) [51] in high-rainfall zones due to smaller property sizes and increased amounts of available feed [52]. One DSE equals the feed required to maintain one 50 kg non-pregnant or lactating castrated male (weather) sheep [51]. There are currently no stocking rate calculation guides for alpacas in Australia. Still, suggestions are that alpacas should be grazed at similar or lower stocking rates compared to sheep due to similar performance or more efficient pasture utilisation of alpacas [11,42]. The ability of the current study to calculate stocking rates in the Australian production system was limited as many producers were unsure how to calculate stocking rates or only owned small numbers where all were run together in one mob.

#### 4.2.6. Veterinary Services

This survey found that the most common reason for visiting a vet was due to birthing complications (43%), followed by external injury (23%). Although respondents were not asked to expand on this, it is reasonable to assume that these are the key areas that require additional expertise or tools beyond what is available on-farm for most producers. From this survey, Australian alpaca producers utilise veterinary services infrequently, with most respondents visiting a vet between 1 and 5 years, with fewer going multiple times a month. However, the frequency of visits did increase as herd size increased. This is to be expected as more animals are to be cared for. When surveying livestock small-holders in Australia, Hernandez-Jover et al. [14] found that where most other livestock species considered veterinarians a valuable source of information, less than half of the surveyed alpaca owners considered veterinarians a helpful information source. Interestingly, a similar viewpoint was taken by SAC owners in Germany [36]. The common feedback from alpaca owners is that few veterinarians have experience or the appropriate knowledge of alpaca or SAC-specific care, basing recommendations on other small ruminant species. The limited use of veterinary services by alpaca producers, based on the low number of visits from survey respondents and the perceived lack of specialised alpaca experience in Australia and overseas, indicates that future training needs to be provided to large animal veterinarians on alpacas and other SAC species to ensure appropriate veterinary care and improve overall animal treatment.

## 5. Conclusions

The Australian alpaca industry largely consists of small to medium-sized herds that keep alpacas as a smallholder farm. The number of commercial alpaca enterprises is increasing, raising concerns about the lack of management guidelines to outline best practices and improve overall herd health and productivity. Further research surrounding paddock behaviour and nutritional requirements in the Australian environment is needed to facilitate the development of industry guidelines and address the producer and veterinary knowledge gap. Addressing these critical knowledge gaps will provide an opportunity to increase the health and productivity of Australian alpaca herds.

## Figures and Tables

**Table 1:** Summary of location (state) and farm scale of survey respondents.

Farm Scale	NSW	QLD	SA	TAS	VIC	WA
Small Scale (≤49 alpacas)	16	7	10	3	13	3
Medium Scale (50–249 alpacas)	13	3	3	0	8	0
Large Scale (≥250 alpacas)	2	1	2	0	3	1
Total	31	11	15	3	24	4
Distribution of Responses (%)	35.23%	12.5%	17.05%	3.41%	27.27%	4.55%

**Table 2:** Primary production reason based on farm scale.

Primary Production Reason	Small Scale	Medium Scale	Large Scale	Total
Commercial production for fibre	6	10	7	23
Guard animals with sheep	2	0	0	2
Other	2	0	0	2
Own as pets	4	0	0	4
Small-scale/smallholder farm	36	12	0	48
Stud/Animal Sales	2	5	2	9

**Table 3:** Effect of production zone and herd size on administering supplements to alpacas based on survey results.

Primary Factors	Sub Factor	*p* Value
Production Zone	Comprehensive Liquid Supplement	0.62
	ADE	0.55
	Selenium	0.89
	Phosphate	0.94
	Basic Liquid Supplement	0.47
	Energised Nutrition Supplement Formulated For Australian Camelids	0.21
	B12	0.61
	No Supplement	0.23
Herd Size	Comprehensive Liquid Supplement	0.96
	ADE	0.37
	Selenium	0.02
	Phosphate	0.19
	Basic Liquid Supplement	0.26
	Energised Nutrition Supplement Formulated For Australian Camelids	1.00
	B12	0.44
	No Supplement	0.10

**Table 4:** First ranked reason for veterinary treatments.

Reason for Veterinary Visit	Number of Respondents
Birthing complications	43.18%
Dental Issues	3.41%
Digestive Issues (bloat, lack of appetite, weight loss)	4.55%
External Injury (e.g., lameness, broken leg, open wound)	22.73%
General husbandry (e.g., vaccinations)	5.68%
Internal Injury (e.g., suspected internal bleeding or trauma)	11.36%
Worms/suspected worms	9.09%

## Data Availability

Data are contained within the article.
